# Associations of Meal Timing and Temporal Eating Patterns with Cardiovascular Disease Among Chinese Older Adults

**DOI:** 10.3390/nu18172778

**Published:** 2026-08-25

**Authors:** Lingfang Wang, Jinghai Li, Jiaming Fang, Shulei Chen, Jingle Peng, Huiqing Ye, Hongying Shi

**Affiliations:** Department of Epidemiology and Health Statistics, School of Public Health, Wenzhou Medical University, Wenzhou 325035, China; wanglingfangzjhz@163.com (L.W.); ljh18368736170@163.com (J.L.); fjmalanam@163.com (J.F.); 15257937648@163.com (S.C.); 13566288052@163.com (J.P.); yehq0918@163.com (H.Y.)

**Keywords:** chrono-nutrition, meal timing, cardiovascular disease, circadian rhythm

## Abstract

**Background**: Direct evidence linking meal timing to cardiovascular disease (CVD) remains limited. This study examined the associations of meal timing and temporal eating patterns with prevalent CVD among Chinese older adults. **Methods**: From September 2021 to October 2025, 1386 community-dwelling adults aged ≥60 years from various socioeconomic regions of China were interviewed face-to-face. Meal timing (breakfast, lunch, and dinner timing) and sleep–wake schedules (wake-up and bedtime) were collected using standardized questionnaires. Four temporal eating parameters were calculated: wake-up to breakfast interval, eating window, dinner to bedtime interval, and the interval from eating midpoint to awake midpoint. Latent profile analysis (LPA) was used to identify temporal eating patterns. Prevalent CVD was assessed by questionnaires and verified with medical records. **Results**: Among 1305 eligible participants (mean [SD] age, 69.6 [6.9] years; 54.6% female), 196 (15.0%) had prevalent CVD. Compared with participants who had breakfast at ≤6:00, those who had breakfast at >7:00 had 75% higher odds of prevalent CVD (95% CI: 1.02, 2.99). Compared with dinner at ≤17:00, dinner at >18:00 had higher odds of prevalent CVD (OR = 2.61, 95% CI: 1.54, 4.43). Three temporal eating patterns were identified: typical eating pattern (58.1%), early-phase eating pattern (10.9%), and late-phase eating pattern (31.0%). Compared with the typical eating pattern, the late-phase eating pattern was associated with 56% higher odds of prevalent CVD (95% CI: 1.08, 2.26), whereas the early-phase eating pattern was associated with 45% lower odds of prevalent CVD (95% CI: 0.28, 1.07), although this association did not reach statistical significance. **Conclusions**: Late-phase eating pattern, particularly delayed breakfast and dinner timing, was associated with increased likelihood of prevalent CVD. Temporal eating patterns may represent a modifiable behavioral target for cardiovascular health in older adults.

## 1. Introduction

Cardiovascular disease (CVD) is one of the leading causes of death and disability worldwide [1], and its risk increases markedly with advancing age [2]. As population aging continues to accelerate globally, the proportion of older adults with CVD has been increasing, imposing substantial economic and caregiving burdens on families and society. According to the latest data from the Global Burden of Disease Study (GBD), the age-standardized incidence rate of CVD among individuals aged 55 years and older in China was 3395.74 per 100,000 people, representing an approximately 20% increase compared with 1990 [3]. Therefore, preventing and reducing the burden of CVD has become an urgent public health priority.

Healthy lifestyle behaviors can effectively reduce CVD risk, with dietary behavior being a critical modifiable factor [4]. However, previous studies have mainly focused on dietary quality [5] and quantity [6], whereas research on dietary timing, particularly the timing of main meals, including breakfast, lunch, and dinner, remains relatively limited [7]. In recent years, research in chrono-nutrition has gradually revealed the impact of the timing of food intake on metabolic health [8]. Early studies found that delaying lunch and dinner timing among young and middle-aged participants may reduce the effectiveness of weight loss and adversely affect cardiometabolic biomarkers, including glucose and lipid metabolism [9,10,11,12,13,14], whereas most of these studies were short-term intervention trials with small sample sizes. Subsequently, larger observational studies explored the associations between meal timing and cardiometabolic risk, but the findings have been inconsistent. Most studies suggested that delayed lunch and dinner timing were associated with increased body weight and blood glucose in adults [15,16,17]; some studies found no association between the timing of main meals and cardiometabolic risk markers in adults [18], while another study showed that delayed breakfast timing was associated with a lower long-term risk of type 2 diabetes among older adults [19]. The inconsistency of existing findings may be related to differences in the life stages of the study populations. Owing to an advanced circadian phase and a greater tendency toward morning chronotype, older adults may have eating times that differ from those of young and middle-aged adults [20]. However, previous studies have rarely accounted comprehensively for circadian-related characteristics, including chronotype, shift-work history, and sleep duration. Inadequate adjustment for these factors may obscure the independent association between meal timing and cardiovascular health. In addition, although the above studies have linked meal timing with cardiometabolic indicators [9,10,11,12,13,14,15,16,17,18], studies directly using CVD as the outcome remain very limited [21].

More importantly, previous chrono-nutrition studies have commonly characterized eating behavior using external clock time, such as breakfast and dinner timing. However, the same clock time may correspond to different biological phases across individuals because of variation in chronotype and sleep–wake schedules. Clock time alone may therefore inadequately reflect the alignment of food intake with an individual’s circadian rhythm [22]. To overcome this limitation, the concept of the circadian timing of food intake has been proposed to characterize meal timing relative to internal biological time rather than to a fixed clock time [23,24]. Measuring the time intervals between eating events and wake-up time, bedtime, or sleep midpoint is considered a reliable approach to assessing the circadian timing of food intake [23]. Supporting its biological relevance, a shorter interval from wake-up to the first meal has been associated with a lower prevalence of obesity [24], whereas longer intervals from dinner to bedtime or to the sleep midpoint have been associated with a lower prevalence of type 2 diabetes and lower body fat levels [25,26]. As eating and sleep behaviors are both closely regulated by the circadian system [27], incorporating indicators of the circadian timing of food intake may provide a more physiologically meaningful characterization of 24 h temporal eating patterns and their associations with CVD.

Latent profile analysis (LPA) is a person-centered statistical approach that can simultaneously consider multiple continuous observed indicators to identify relatively homogeneous population subgroups with similar behavioral patterns [28]. Despite these potential advantages, the application of this approach in chrono-nutrition research remains limited. To date, only one study has used LPA to explore temporal eating patterns based on indicators of circadian timing of food intake and sleep health among U.S. adults [27]. To our knowledge, no previous study has examined the association between LPA-derived temporal eating patterns and CVD, and such patterns have not been systematically characterized among Chinese older adults.

Accordingly, this study aimed to examine the independent associations between the meal timing and prevalent CVD among Chinese older adults after controlling for circadian rhythm-related confounders. We further identified temporal eating patterns using LPA based on multidimensional indicators of circadian food intake timing and investigated the associations between these patterns and prevalent CVD. This study may provide evidence for optimizing dietary behaviors to improve cardiovascular health in older adults.

## 2. Materials and Methods

### 2.1. Study Design and Participants

This cross-sectional study was conducted from September 2021 to October 2025 among community-dwelling adults aged 60 years and older from regions with different economic levels in China. A total of 1386 participants were initially recruited from 13 regions across Zhejiang, Anhui, Guizhou, Jiangxi, and Henan provinces, using multistage stratified sampling method [29,30]. Face-to-face interviews were conducted to collect information on demographic characteristics, health status, lifestyle factors, dietary behaviors, and circadian rhythm-related variables. Comprehensive quality control measures were implemented throughout the entire survey process [29,30]. The study protocol was approved by the Ethics Committee of Wenzhou Medical University (Approval No.: 2020-060), and all participants signed the informed consent form.

Participants were eligible if they were aged 60 years or older and had normal verbal expression and communication abilities. Based on previously reported prevalence estimates of CVD among Chinese older adults (26.0–31.2%) [31,32], with α set at 0.05 (two-sided), an odds ratio of 0.55–0.70 [33], and a power of 0.80, the required sample size was estimated to be 770–1132 participants. Assuming a non-response rate of 10%, the final required sample size was 855–1258 participants. After excluding participants with missing information on prevalent CVD (*n* = 1), missing information on meal timing (*n* = 78), atypical breakfast time (e.g., before 4:00 or after 10:00, *n* = 2) [29], 1305 older adults were included in the final statistical analysis.

### 2.2. Evaluation of Meal Timing and Temporal Eating Patterns

Temporal eating behaviors were assessed using a structured questionnaire developed based on the theoretical framework of chrono-nutrition. The questionnaire covered core domains including meal timing, meal frequency, meal regularity, and daily schedule timing. Meal timing was assessed using the question, “During the past 12 months, what time did you usually have breakfast (lunch and dinner)?” Sleep timing was assessed using the question, “During the past 12 months, what time did you usually go to bed and wake up?” A validation study was conducted for the activity timing variables in the questionnaire, including wake-up time, breakfast timing, lunch timing, dinner timing, and bedtime. The test–retest reliability ranged from 0.72 to 0.78, and the validity coefficients, using a 7-day activity diary as the reference standard, ranged from 0.72 to 0.87, indicating good reliability and validity of the questionnaire [30]. Because there is currently no universally accepted standard definition of “breakfast,” “lunch,” and “dinner,” we did not classify the three main meals using specific clock-time cutoffs [34].

To reflect the degree of alignment between food intake and circadian rhythms, we further calculated several timing-related variables based on the above responses. These included the interval from wake-up to breakfast, the interval from dinner to bedtime, the interval from breakfast to dinner (i.e., daily eating window), the midpoint between breakfast and dinner (i.e., eating midpoint), the midpoint between wake-up time and bedtime (i.e., awake midpoint), and the interval between the eating midpoint and the awake midpoint [20,35]. These variables were then used in LPA to identify temporal eating patterns.

### 2.3. Evaluation of Prevalent CVD

Prevalent CVD was defined based on previous large-scale epidemiological studies [36,37]. CVD status was initially determined from participants’ self-reported history of physician-diagnosed conditions and was subsequently verified using relevant medical records. Specifically, participants were asked, “Have you ever been diagnosed with coronary heart disease, including angina pectoris and myocardial infarction?” and “Have you ever been diagnosed with stroke?” In addition, information on other cardiovascular conditions, such as heart failure, was collected in the chronic disease history module. Participants who reported a previous diagnosis of coronary heart disease, including angina pectoris and myocardial infarction, stroke, or heart failure were classified as having prevalent CVD [36,37]. Relevant medical records, including outpatient medical records, examination reports, or hospitalization records, were reviewed during the interview to verify the reported CVD history and were compared with the corresponding questionnaire responses. The medical records were reviewed by trained members of the research team. When questionnaire responses were inconsistent with the available medical documentation, or when the diagnosis remained uncertain, participants were contacted for additional confirmation.

### 2.4. Assessment of Covariates and Potential Intermediate Factors

Based on previous studies on CVD and chrono-nutrition, the covariates included general demographic characteristics, lifestyle factors, dietary factors, and circadian rhythm-related factors, whereas metabolic factors were additionally considered as potential intermediate factors.

Demographic characteristics included age (continuous), sex (male, female), marital status (married, divorced/widowed/never married), community type (urban, rural), annual household income (<20,000 RMB, 20,000–49,999 RMB, ≥50,000 RMB), education level (illiterate, primary school or below, junior high school or above), survey period (year), and family history of CVD (yes, no). Family history of CVD was defined as a history of coronary heart disease or stroke among participants’ first-degree relatives.

Lifestyle factors included smoking status (never, former, current), drinking status (never, former, current), physical activity (low, moderate, high). Physical activity was measured using the Chinese version of the International Physical Activity Questionnaire-Short Form (IPAQ-SF). Walking, moderate-intensity physical activity, and vigorous-intensity physical activity were assigned metabolic equivalent values of 3.3, 4.0, and 8.0 METs, respectively. Total weekly metabolic equivalent minutes were calculated as follows: physical activity (MET-min/wk) = MET × weekly frequency × duration of each activity. Participants were categorized into three physical activity levels: low, moderate, and high [38].

Dietary factors included meal frequency (continuous), satiety (continuous), and diet quality (high, low). Meal frequency was assessed based on self-reported eating occasions across the day, including before breakfast, at breakfast, between breakfast and lunch, at lunch, between lunch and dinner, at dinner, after dinner and before bedtime, and after falling asleep [39]. Satiety was used as an indicator of individual energy intake and was scored from 1 to 10, with higher scores indicating greater satiety [40]. Diet quality was assessed using the established dietary scoring method from the China Kadoorie Biobank (CKB). The score was constructed based on the intake of five food groups (fresh vegetables, fresh fruits, red meat, legumes, and fish), with one point assigned for each component when the recommended intake criterion was met. The criteria included daily consumption of fresh vegetables and fruits, red meat consumption on 1–6 days per week, legumes consumption on ≥4 days per week, and fish consumption on ≥1 day per week. The total score ranged from 0 to 5, with scores of 4–5 classified as high diet quality and scores < 4 classified as low diet quality [41].

Circadian rhythm-related factors included sleep duration, sleep quality, chronotype, and shift work history. Nighttime sleep duration was assessed using the question, “During the past month, how many hours of actual sleep did you get at night?” and was categorized into three groups: <6, 6–8, and >8 h [42]. Sleep quality was assessed using the Chinese version of the Pittsburgh Sleep Quality Index (PSQI). A PSQI score > 7 was defined as poor sleep quality, whereas a score ≤ 7 was defined as good sleep quality [43]. Chronotype was assessed using the reduced Morningness-Eveningness Questionnaire-5 (rMEQ-5) and categorized into five groups: definite evening type (4–7 points), moderate evening type (8–11 points), intermediate type (12–17 points), moderate morning type (18–21 points), and definite morning type (22–25 points) [44].

Metabolic factors included body mass index (BMI) (<24.00 kg/m^2^, ≥24.00 kg/m^2^), diabetes (yes, no), and hypertension (yes, no). BMI was calculated by dividing weight (kg) by height squared (m^2^) and categorized into non-overweight (<24.00 kg/m^2^) and overweight or above (≥24.00 kg/m^2^) [45].

### 2.5. Statistical Analysis

Continuous variables were expressed as means ± standard deviations (SD) or medians (IQR), and categorical variables were expressed as frequencies and percentages. Basic characteristics of older adults with different temporal eating patterns were compared using one-way analysis of variance for normally distributed continuous variables, the Kruskal–Wallis test for non-normally distributed continuous or ordinal variables, and the chi-square test for categorical variables.

To identify temporal eating patterns among older adults, LPA was performed based on the interval from wake-up to breakfast, the interval from dinner to bedtime, the eating window, and the interval between the eating midpoint and the awake midpoint [27]. Models with 2 to 6 classes were fitted, and the optimal number of profiles was selected by comprehensively considering model fit indices, interpretability, and practical applicability [27]. The criteria included: (1) the Bayesian information criterion (BIC) and Akaike information criterion (AIC), with lower values indicating better model fit; (2) likelihood ratio tests, including the Vuong–Lo–Mendell–Rubin likelihood ratio test (VLMR-LRT) and the bootstrap likelihood ratio test (BLRT), which were used to assess whether the *k*-class model provided a significant improvement over the *k*-1-class model; (3) entropy, with a value ≥0.8 indicating relatively accurate classification; and (4) a minimum class proportion of at least 5% to ensure adequate statistical power.

Restricted cubic splines (RCS) were used to explore the shape of the associations between meal timing, including breakfast, lunch, and dinner timing, and prevalent CVD. The number of knots was determined based on the minimum AIC, and three knots were selected for the final models [46]. The knots were located at the 10th, 50th, and 90th percentiles of each meal timing variable. Logistic regression models were used to examine the independent associations of meal timing and temporal eating patterns with prevalent CVD, and the results were expressed as odds ratios (OR) and 95% confidence intervals (CI). Meal timing was analyzed both as a continuous variable and as a categorical variable. After nonlinear associations were excluded based on the RCS curves, meal timing was categorized into tertiles according to approximate tertile values with reference to previous study [37]. The *P* for trend was obtained by entering the median value of each meal timing category into the model as a continuous variable. Model I was an unadjusted model. Model II was adjusted for demographic characteristics, including age, sex, education level, community type, annual household income, marital status, family history of CVD, and survey period. Model III, designated as the primary model, further controlled for lifestyle factors (smoking status, drinking status, and physical activity), dietary factors (diet quality, satiety, and meal frequency), and circadian rhythm-related factors (sleep duration, chronotype, sleep quality, and shift work history). However, in analyses of the temporal eating patterns, chronotype was not included in Model III because wake-up time and bedtime had already been used to derive the temporal eating-pattern indicators. Additional adjustment for chronotype might therefore result in potential over-adjustment. Model IV was an additional adjustment model that further included BMI, diabetes, and hypertension, which may represent potential intermediate factors or established cardiovascular risk factors. Multicollinearity diagnostics were performed for all covariates, and the variance inflation factor (VIF) was <5. The complete case analysis was applied for all regression models, and participants with missing values in any covariates (0.15–1.84%) were excluded from the corresponding analyses [47]. Exploratory analyses were further conducted to examine the associations of meal timing and temporal eating patterns with specific CVD subtypes. Due to the extremely limited number of heart failure cases (*n* = 2), subtype analyses were restricted to coronary heart disease and stroke.

In addition, to examine whether the associations of meal timing and temporal eating patterns with prevalent CVD were consistent across older adults with different characteristics, subgroup analyses were performed according to age, sex, smoking status, drinking status, and other characteristics. *p* values for multiplicative interaction were calculated using likelihood ratio tests. Subgroup analyses and interaction analyses were conducted as exploratory analyses.

To verify the robustness of the results, sensitivity analyses were performed. The study population was restricted to participants who were definite or moderate morning types, had no history of shift work, and had no family history of CVD to assess whether the shape and direction of the associations changed. To evaluate the potential influence of late-night snacking, participants who reported a late-night snack time were excluded (*n* = 19), and the LPA and subsequent analyses of the association with prevalent CVD were repeated among the remaining participants. In addition, E-value were calculated to evaluate the potential impact of unmeasured confounding on the results [48]. R 4.4.3 (R Foundation for Statistical Computing, Vienna, Austria) was used for all analyses and visualizations. Statistical significance was set at *p* < 0.05.

## 3. Results

Among the 1305 participants, the mean age was 69.6 ± 6.9 years, and 592 participants were male (45.4%). The mean timings of main meals and daily schedule events were as follows: wake-up time (05:47 ± 00:58), breakfast timing (06:53 ± 00:52), lunch timing (11:34 ± 00:33), dinner timing (17:40 ± 00:49) and bedtime (21:00 ± 01:18). A total of 196 participants (15.0%) reported having prevalent CVD, including 95 men (16.1%) and 101 women (14.2%). Regarding the distribution of CVD subtypes, 121 participants had coronary heart disease, 96 had stroke, and 2 had heart failure.

### 3.1. Distribution of Temporal Eating Patterns Among Older Adults

**Figure 1:**
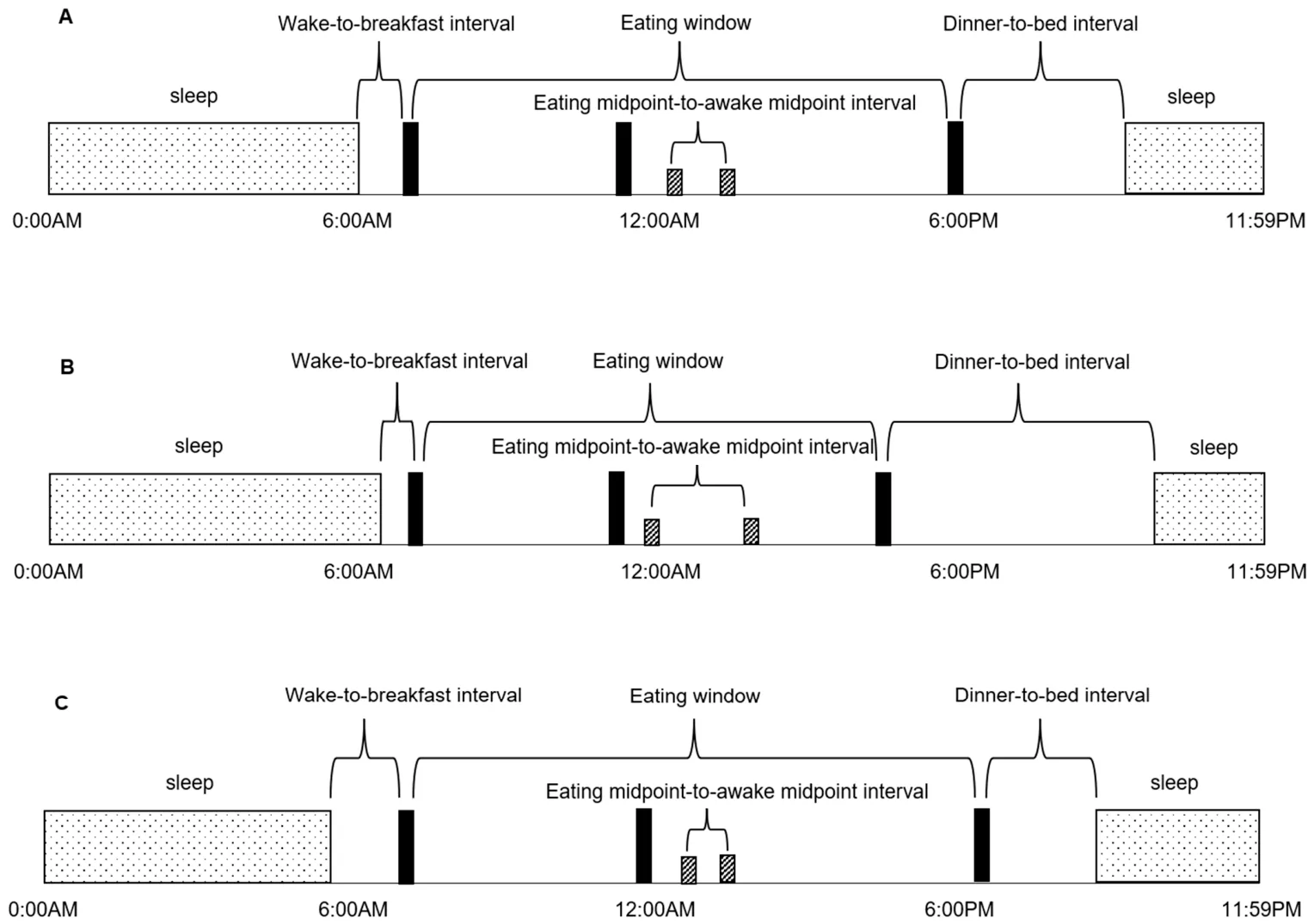
Meal timing variables across the three temporal eating patterns. Solid black bars indicate eating activities, dashed bars indicate sleep, and shaded bars indicate the eating midpoint and awake midpoint. Panel (**A**) represents the typical eating pattern, in which all timing-related variables approximated the mean levels of the overall study population. Panel (**B**) represents the early-phase eating pattern, characterized by a shorter interval from wake-up to breakfast, a shorter eating window, a longer interval from dinner to bedtime, and a longer interval between the eating midpoint and the awake midpoint. Panel (**C**) represents the late-phase eating pattern, characterized by a longer interval from wake-up to breakfast, a longer eating window, a shorter interval from dinner to bedtime, and a shorter interval between the eating midpoint and the awake midpoint.

Based on LPA, three temporal eating patterns were identified: the typical eating pattern (*n* = 758), early-phase eating pattern (*n* = 142), and late-phase eating pattern (*n* = 405) (Tables S1 and S2 and Figure 1). The three-class model was selected because it showed good classification quality (entropy ≥ 0.8), relatively lower AIC and BIC values, and was the only model in which all latent classes exceeded the predefined minimum proportion of 5% (Table S1). The typical eating pattern was characterized by breakfast occurring approximately 1 h after wake-up, dinner ending 3.7 h before bedtime, an eating window of 10.7 h, and an eating midpoint 1.3 h earlier than the awake midpoint. These timing-related variables were very close to the overall population averages (e.g., eating window of 10.8 h and an eating midpoint 1.1 h before the awake midpoint), representing a relatively conventional eating rhythm. The early-phase eating pattern was characterized by breakfast occurring approximately 0.8 h after wake-up, an eating window of 10.1 h, dinner ending 5.7 h before bedtime, and an eating midpoint 2.4 h earlier than the awake midpoint, representing an earlier eating rhythm. The late-phase eating pattern was characterized by breakfast occurring 1.4 h after wake-up, an eating window of 11.3 h, dinner ending 1.8 h before bedtime, and an eating midpoint 0.2 h earlier than the awake midpoint, indicating an overall delayed eating rhythm.

### 3.2. Basic Characteristics of Older Adults by Temporal Eating Patterns

Table 1 presents the differences in basic characteristics among older adults with different temporal eating patterns. Compared with those with the typical eating pattern, older adults with the early-phase eating pattern had higher proportions of urban residents, individuals with higher education levels, married individuals, those with middle income, and those with a family history of CVD. They also had shorter nighttime sleep duration, better sleep quality, and a higher proportion of moderate morning types. In contrast, older adults with the late-phase eating pattern had higher proportions of rural residents, individuals with lower education levels, non-married individuals, those with low income, and current drinkers. They also had longer nighttime sleep duration, poorer sleep quality, and the highest proportion of definite morning types. All differences were statistically significant (*p* < 0.05). In addition, basic characteristics of the study participants were compared according to prevalent CVD status and meal timing categories, as shown in Tables S3 and S4.

### 3.3. Associations of Meal Timing with Prevalent CVD Among Older Adults

Restricted cubic spline (RCS) curves were first used to preliminarily explore the associations between meal timing and prevalent CVD (Figure S1). No nonlinear associations were observed between meal timing and prevalent CVD (*P* for nonlinear > 0.05). Therefore, meal timing was included in the models both as a continuous variable and as a categorical variable to examine its independent association with prevalent CVD among older adults. The results showed that later meal timing was associated with a higher prevalence of CVD.

In the primary model (Model III), which was adjusted for sociodemographic characteristics, lifestyle factors, dietary factors, and circadian rhythm-related factors. Each 1 h delay in breakfast timing was associated with 30% higher odds of CVD among older adults (95% CI: 1.04, 1.62); compared with participants with breakfast timing ≤ 6:00, those with breakfast timing > 7:00 had 75% higher odds of prevalent CVD (95% CI: 1.02, 2.99). Similarly, each 1 h delay in dinner timing was associated with 41% (95% CI: 1.11, 1.79) higher odds of prevalent CVD. Participants with dinner timing > 18:00, compared with those with dinner timing ≤ 17:00, had higher odds of prevalent CVD, with an OR (95% CI) of 2.61 (1.54, 4.43). However, for lunch timing, compared with participants who had lunch at ≤11:00, those with lunch at 11:00–12:00 and >12:00 had ORs (95% CIs) of 1.40 (0.93, 2.10) and 1.56 (0.79, 3.08), respectively, although neither association reached statistical significance (Table 2).

After further adjustment for metabolic factors, including BMI, diabetes, and hypertension, in Model IV, the associations of breakfast and dinner timing with prevalent CVD remained generally consistent with those observed in the primary model (Model III). Each 1 h delay in breakfast and dinner timing was associated with 28% (95% CI: 1.02, 1.60) and 47% (95% CI: 1.15, 1.88) higher odds of prevalent CVD, respectively. These findings suggest that the observed associations were not substantially attenuated after accounting for these potential intermediate factors. The association between lunch timing and prevalent CVD remained weak and was not statistically significant, with an OR of 1.35 (95% CI: 0.97, 1.88) per 1 h delay in lunch timing (Table 2).

In addition, exploratory analyses of the associations between meal timing and prevalent CVD subtypes showed that, in the model III, each 1 h delay in breakfast timing was associated with 43% higher odds of stroke (95% CI: 1.06, 1.93), and each 1 h delay in lunch timing was associated with 47% higher odds of coronary heart disease (CHD) (95% CI: 1.00, 2.15). Compared with participants with dinner timing ≤ 17:00, those with dinner timing > 18:00 had higher odds of both coronary heart disease and stroke, with ORs (95% CIs) of 2.55 (1.33, 4.91) and 2.90 (1.44, 5.83), respectively. Detailed results are shown in Tables S5–S7.

### 3.4. Associations of Temporal Eating Patterns with Prevalent CVD Among Older Adults

In the primary model (Model III), compared with the typical eating pattern, the early-phase eating pattern was associated with 45% lower odds of prevalent CVD (95% CI: 0.28, 1.07), although this association did not reach statistical significance. In contrast, the late-phase eating pattern was associated with 56% higher odds of prevalent CVD (95% CI: 1.08, 2.26). In the additional model (Model IV), the associations between temporal eating patterns and prevalent CVD were generally similar to those observed in the primary model, as shown in Table 3.

Exploratory analyses of the associations between temporal eating patterns and prevalent CVD subtypes showed that, in the primary model, compared with the typical eating pattern, the early-phase eating pattern was associated with 63% lower odds of CHD (95% CI: 0.15, 0.91), and the late-phase eating pattern was associated with an OR of 2.08 (95% CI: 1.28, 3.38) for stroke. Detailed results are shown in Table S8. However, these subtype-specific findings should be interpreted cautiously because some CVD subtypes included a limited number of cases.

### 3.5. Exploratory Subgroup Analysis

Exploratory subgroup analyses of the association between meal timing and prevalent CVD showed that the association of later dinner timing (>18:00) with prevalent CVD was more pronounced among men, with OR (95% CI) of 3.49 (1.45, 8.42). The interaction tests were statistically significant (*P* for interaction < 0.05). In contrast, the associations between dinner timing and prevalent CVD were generally consistent across subgroups defined by age, smoking status, drinking status, physical activity level, BMI, sleep duration, and sleep quality (*P* for interaction > 0.05). In addition, the associations of breakfast and lunch timing with prevalent CVD were robust across all subgroups (*P* for interaction > 0.05) (Tables S9–S11).

Exploratory subgroup analyses showed that, among short and long sleepers, the associations of both early-phase and late-phase eating patterns with prevalent CVD appeared more pronounced, with ORs (95% CIs) of 0.07 (0.01, 0.55) and 1.83 (1.02, 3.28), respectively. The interaction test was statistically significant (*P* for interaction < 0.05). In contrast, the associations between temporal eating patterns and prevalent CVD were generally consistent across subgroups defined by age, sex, smoking status, drinking status, physical activity level, BMI, and sleep quality (*P* for interaction > 0.05) (Table S12). Given the exploratory nature of these analyses, the subgroup findings should be interpreted cautiously because some strata contained relatively few prevalent CVD cases, which may have resulted in unstable estimates and wide confidence intervals.

### 3.6. Sensitivity Analysis

Several sensitivity analyses were performed to examine the robustness of the study findings. First, considering that the study population mainly consisted of individuals with definite or moderate morning chronotype and no history of shift work, we repeated the analyses after restricting the study population to these subgroups. The results remained robust (Tables S13–S16). Second, to further minimize potential confounding due to genetic susceptibility, we conducted additional restricted analyses among participants without a family history of CVD, and the results were still consistent with the main analyses (Tables S13–S16). Third, after excluding participants with a habit of late-night snacking, the identified temporal eating profiles and their associations with prevalent CVD remained largely consistent with the main findings (Table S17). Finally, to assess the potential impact of unmeasured confounding, E-values were calculated. The E-values for the associations of breakfast timing and dinner timing, analyzed as continuous variables, with prevalent CVD were 1.92 (lower limit of the 95% CI, 1.24) and 4.66 (lower limit of the 95% CI, 2.45), respectively. In addition, compared with the typical eating pattern, the E-value for the late-phase eating pattern was 2.49 (lower limit of the 95% CI, 1.37). Although some associations showed relatively large E-values, the E-values for the lower confidence limits suggest that residual confounding cannot be completely excluded (Figure S2).

## 4. Discussion

To our knowledge, this study is the first to explore the association between meal timing and prevalent CVD among Chinese older adults. The results suggested linear associations between meal timing and prevalent CVD, with later breakfast and dinner timing associated with higher odds of prevalent CVD. In addition, based on indicators reflecting the circadian timing of food intake, this study used LPA to identify temporal eating patterns among older adults, further providing complementary evidence: compared with the typical eating pattern, participants with the late-phase eating pattern had significantly higher odds of prevalent CVD. Notably, the associations of meal timing and temporal eating patterns with prevalent CVD were generally similar after further adjustment for BMI, diabetes, and hypertension. This suggests that these potential intermediate metabolic factors may not fully explain the observed associations, although their mediating roles cannot be established in this cross-sectional study.

Most previous epidemiological studies have shown that delayed meal timing is unfavorable for cardiovascular health. A 1-year prospective study of 116 women in the United States found that a later first-meal timing was associated with a lower cardiovascular health score, as well as higher diastolic blood pressure and fasting glucose levels [49]. Another study using cross-sectional data from the 2005–2016 National Health and Nutrition Examination Survey also showed that later first- and last-meal timing was associated with cardiometabolic risk markers [50]. In addition, a cross-sectional study based on the Henan Rural Cohort in China found that delayed dinner timing was associated with reduced insulin sensitivity [17]. The outcomes of these studies were all cardiovascular risk indicators. In contrast, the large-scale French NutriNet-Santé cohort study directly examined the association between meal timing and CVD and showed that each 1 h delay in the first meal was associated with a 6% higher risk of overall CVD, whereas each 1 h delay in the last meal was associated with an 8% higher risk of cerebrovascular disease [37]. In line with previous studies, our results suggest that delayed meal timing may be unfavorably associated with CVD.

This study also identified temporal eating patterns reflecting the circadian rhythm characteristics of older adults and found that the late-phase eating pattern was associated with higher odds of CVD. Recent evidence suggests that, in addition to the duration of the eating window, the position of the eating window relative to biological time is also important for metabolic health. One study found that adherence to early time-restricted eating improved anthropometric measures, insulin resistance, and blood glucose levels [51]. Another study showed that, compared with a short eating window starting in the morning, a long eating window extending into the evening was particularly detrimental to glucose and lipid metabolism and the expression of circadian clock genes [52]. These previous findings further support our results, suggesting that starting and ending food intake earlier during the waking period may help reduce the likelihood of CVD.

Although previous studies have identified associations between chrono-nutrition behavior and cardiometabolic risk, the underlying biological mechanisms have not been fully elucidated. First, food intake timing is a key zeitgeber that regulates the phase of peripheral clocks [53]. Late eating may disrupt the synchronization between the circadian system and the external environment [54], leading to persistent circadian disruption and thereby potentially increasing the risk of chronic diseases [55]. Second, circadian variation in glucose and lipid metabolism may represent an important explanatory pathway. In general, glucose tolerance, insulin sensitivity, and lipid metabolic capacity are higher during the daytime and decline at night [56]. Crossover trials have shown that late eating may lead to postprandial hyperglycemia, impaired glucose tolerance, and reduced fat oxidation [57,58], thereby increasing cardiometabolic burden. On this basis, metabolic dysfunction may further induce chronic low-grade inflammation, promote vascular endothelial dysfunction, and accelerate the progression of atherosclerosis [59]. Third, delayed meal timing may affect the circadian secretion of leptin, an adipocyte-derived hormone involved in appetite suppression and energy balance; altered leptin rhythms may contribute to obesity and related metabolic disorders, including CVD and diabetes [60,61]. Fourth, circadian variation in energy expenditure may also be an important mechanism. Studies have shown that, under conditions of identical caloric and nutrient intake, diet-induced thermogenesis (DIT) is significantly higher in the morning than in the evening [62]. This suggests that consuming food earlier in the day may help maintain metabolic homeostasis by increasing energy expenditure and reducing fat accumulation. Overall, the influence of food intake timing on cardiometabolic health is a complex process involving interactions among multiple physiological systems.

To our knowledge, this is the first study to investigate the association between meal timing and prevalent CVD among Chinese older adults. By carefully accounting for potential confounding from circadian rhythm-related factors, including sleep quality, chronotype, and shift work history, this study enhanced the reliability of the findings. Furthermore, it is also the first to characterize temporal eating patterns in Chinese older adults and examine their associations with prevalent CVD.

This study has several limitations. First, the cross-sectional design precludes causal inference. Although meal and sleep timing were assessed based on participants’ usual behaviors during the previous 12 months, CVD was defined based on a history of previous physician diagnosis. Therefore, CVD may have preceded the reported eating patterns in some participants, and reverse causation cannot be completely excluded. Second, although all prevalent CVD cases were verified using available medical documentation, standardized ICD codes were not uniformly available across the medical records and therefore were not used for disease classification. Consequently, some degree of outcome misclassification cannot be completely excluded. Third, the collection of chrono-nutrition variables relied on participants’ self-report and may therefore be subject to misreporting and recall bias. However, our validation study of the dietary and sleep behavior questionnaire showed that these chrono-nutrition variables had good reliability and validity. Fourth, for feasibility reasons, this study did not use more accurate methods, such as 24 h dietary recalls, to assess and control for the confounding effect of total energy intake. However, based on previous studies, we adjusted for satiety level as a proxy variable for energy intake. Future studies may use more precise tools to assess caloric intake. Fifth, although the indicators used for LPA were selected based on previous chrono-nutrition research and our conceptual framework, the temporal eating profiles may still be influenced by indicator selection. Different combinations of timing variables may capture different dimensions of temporal eating behavior and potentially lead to different profile solutions. Future studies incorporating objective assessments of circadian timing, such as dim-light melatonin onset (DLMO), together with more detailed measurements of eating behavior, are needed to further validate these temporal eating patterns. Sixth, despite adjustment for a series of established confounders, residual confounding may still exist due to unmeasured or imperfectly measured factors (e.g., dietary composition and energy distribution). E-values were calculated to evaluate the potential impact of unmeasured confounding; however, they cannot completely eliminate residual confounding or address other sources of bias, such as reverse causation and measurement error. Finally, the study population was limited to Chinese older adults, and the generalizability of the findings to other cultural or social contexts requires further validation.

## 5. Conclusions

Based on data from community-dwelling older adults across multiple regions of China, this study systematically examined the associations of meal timing (external clock time) and temporal eating patterns with prevalent CVD. The main findings were as follows: later breakfast and dinner times, particularly breakfast after 7:00 and dinner after 18:00, were associated with a higher likelihood of prevalent CVD, whereas no significant association was observed for lunch timing. LPA further identified three temporal eating patterns. Among them, the late-phase eating pattern was associated with significantly higher odds of prevalent CVD compared with the typical eating pattern, whereas the early-phase eating pattern showed a trend toward lower odds, although this association did not reach statistical significance. The two analytical perspectives, meal timing and temporal eating patterns, complement each other and jointly suggest that earlier timing of food intake during the waking period may be associated with cardiovascular health among older adults. Future prospective and intervention studies in more diverse populations are needed to further validate these findings and to confirm the long-term benefits of chrono-nutrition strategies in precision metabolic management.

## Figures and Tables

**Table 1:** Characteristics of older adults by temporal eating patterns.

Characteristics	Meal Timing Patterns			*p* Value
	Typical Eating	Early-Phase Eating	Late-Phase Eating	
	(*n* = 758)	(*n* = 142)	(*n* = 405)	
Age, years	69.6 ± 6.9	68.6 ± 6.5	70.0 ± 6.9	0.100
Female, *n* (%)	432 (57.0)	77 (54.2)	204 (50.4)	0.096
Residence, *n* (%)				<0.001
Urban	176 (23.2)	42 (29.6)	45 (11.1)	
Rural	582 (76.8)	100 (70.4)	360 (88.9)	
Education level, *n* (%)				0.031
Illiterate	197 (26.0)	35 (24.7)	110 (27.2)	
Primary school	313 (41.3)	55 (38.7)	194 (47.9)	
Junior high school or above	248 (32.7)	52 (36.6)	101 (24.9)	
Marital status, *n* (%)				0.007
Married	643 (84.8)	121 (85.2)	315 (77.8)	
Divorced/widowed/never married	115 (15.2)	21 (14.8)	90 (22.2)	
Annual household income, *n* (%) ^1^				<0.001
<20,000 RMB	155 (20.7)	25 (17.7)	120 (30.6)	
20,000–49,999 RMB	310 (41.4)	70 (49.7)	155 (39.5)	
≥50,000 RMB	283 (37.8)	46 (32.6)	117 (29.9)	
Diabetes, *n* (%)				0.479
No	637 (84.0)	125 (88.0)	342 (84.4)	
Yes	121 (16.0)	17 (12.0)	63 (15.6)	
Hypertension, *n* (%)				0.703
No	382 (50.4)	77 (54.2)	206 (50.9)	
Yes	376 (49.6)	65 (45.8)	199 (49.1)	
Family history of CVD, *n* (%) ^1^				0.037
No	693 (92.3)	119 (85.6)	359 (90.4)	
Yes	58 (7.7)	20 (14.4)	38 (9.6)	
Smoking status, *n* (%) ^1^				0.088
Never smoker	557 (73.6)	97 (68.3)	268 (66.3)	
Former smoker	100 (13.2)	19 (13.4)	65 (16.1)	
Current smoker	100 (13.2)	26 (18.3)	71 (17.6)	
Drinking status, *n* (%) ^1^				0.004
Never drinker	522 (69.0)	96 (67.6)	240 (59.4)	
Former drinker	92 (12.2)	16 (11.3)	48 (11.9)	
Current drinker	143 (18.9)	30 (21.1)	116 (28.7)	
Physical activity level, *n* (%) ^1^				0.308
Low	121 (16.0)	23 (16.3)	75 (18.6)	
Moderate	339 (44.8)	56 (39.7)	156 (38.7)	
High	297 (39.2)	62 (44.0)	172 (42.7)	
Body mass index, *n* (%) ^1^				0.264
<24.00 kg/m^2^	443 (58.5)	73 (51.4)	237 (58.7)	
≥24.00 kg/m^2^	314 (41.5)	69 (48.6)	167 (41.3)	
Diet quality, *n* (%) ^1^				0.094
Low	638 (84.7)	117 (83.0)	352 (88.9)	
High	115 (15.3)	24 (17.0)	44 (11.1)	
Satiety ^1^	7.84 ± 1.01	7.72 ± 1.01	7.86 ± 1.01	0.253
Meal frequency ^1^	3.11 ± 0.35	3.16 ± 0.45	3.08 ± 0.32	0.056
Sleep duration, *n* (%)				0.001
<6 h	125 (16.5)	36 (25.4)	81 (20.0)	
6–8 h	512 (67.6)	78 (54.9)	232 (57.3)	
>8 h	121 (16.0)	28 (19.7)	92 (22.7)	
Sleep quality, *n* (%) ^1^				0.034
Good	546 (72.8)	111 (78.2)	271 (67.6)	
Poor	204 (27.2)	31 (21.8)	130 (32.4)	
Chronotype, *n* (%) ^1, 2^				<0.001
Intermediate type	63 (8.4)	33 (23.2)	36 (9.0)	
Moderately morning type	340 (45.1)	78 (54.9)	130 (32.5)	
Definite morning type	351 (46.6)	31 (21.8)	234 (58.5)	
Shift work, *n* (%) ^1^				0.440
No	632 (84.0)	114 (80.9)	340 (85.4)	
Yes	120 (16.0)	27 (19.2)	58 (14.6)	

^1^ Missing data: annual household income (*n* = 24, 1.84%), family history of CVD (*n* = 18, 1.38%), smoking status (*n* = 2, 0.15%), drinking status (*n* = 2, 0.15%), physical activity level (*n* = 4, 0.31%), BMI (*n* = 2, 0.15%), diet quality (*n* = 15, 1.15%), satiety (*n* = 17, 1.3%), meal frequency (*n* = 14, 1.07%), sleep quality (*n* = 12, 0.92%), chronotype (*n* = 9, 0.69%), shift work (*n* = 14, 1.07%). ^2^ The definite evening and moderate evening chronotype categories were not presented because no participants were classified into these categories.

**Table 2:** Associations between meal timing and prevalent CVD among older adults.

Exposure	No.	CVD, *n* (%)	OR (95% CI)			
			Model I ^a^	Model II ^b^	Model III ^c^	Model IV ^d^
CVD cases/total N			196/1305	193/1272	189/1237	189/1236
Breakfast time						
Continuous	1305	196 (15.0)	1.21 (1.02, 1.45)	1.30 (1.07, 1.57)	1.30 (1.04, 1.62)	1.28 (1.02, 1.60)
Categorical ^e^						
≤6:00	283	33 (11.7)	Ref.	Ref.	Ref.	Ref.
6:00–7:00	589	90 (15.3)	1.37 (0.89, 2.09)	1.51 (0.96, 2.39)	1.44 (0.88, 2.36)	1.38 (0.84, 2.27)
>7:00	433	73 (16.9)	1.54 (0.99, 2.39)	1.83 (1.13, 2.96)	1.75 (1.02, 2.99)	1.70 (0.99, 2.94)
*P* for trend			0.065	0.012	0.045	0.056
Lunch time						
Continuous	1305	196 (15.0)	1.13 (0.86, 1.49)	1.28 (0.95, 1.73)	1.28 (0.93, 1.78)	1.35 (0.97, 1.88)
Categorical ^e^						
≤11:00	366	51 (13.9)	Ref.	Ref.	Ref.	Ref.
11:00–12:00	829	125 (15.1)	1.10 (0.77, 1.56)	1.25 (0.86, 1.81)	1.40 (0.93, 2.10)	1.43 (0.95, 2.18)
>12:00	110	20 (18.2)	1.37 (0.78, 2.42)	1.69 (0.91, 3.16)	1.56 (0.79, 3.08)	1.74 (0.87, 3.49)
*P* for trend			0.313	0.090	0.102	0.059
Dinner time						
Continuous	1305	196 (15.0)	1.07 (0.89, 1.29)	1.31 (1.05, 1.62)	1.41 (1.11, 1.79)	1.47 (1.15, 1.88)
Categorical ^e^						
≤17:00	461	62 (13.5)	Ref.	Ref.	Ref.	Ref.
17:00–18:00	604	88 (14.6)	1.10 (0.77, 1.56)	1.24 (0.84, 1.81)	1.23 (0.82, 1.86)	1.31 (0.86, 1.99)
>18:00	240	46 (19.2)	1.53 (1.00, 2.32)	2.31 (1.43, 3.74)	2.61 (1.54, 4.43)	3.06 (1.78, 5.28)
*P* for trend			0.062	0.001	0.001	<0.001

^a^ Model I was an unadjusted model. ^b^ Model II adjusted for age, sex, education level, community type, annual household income, marital status, family history of CVD, and survey period; ^c^ Model III was the primary model and additionally adjusted for smoking status, drinking status, physical activity, diet quality, meal frequency, satiety, sleep duration, sleep quality, chronotype, and shift work based on Model II; ^d^ Model IV was an additional adjustment model and was further adjusted for metabolic factors, including BMI, diabetes, and hypertension based on Model III. ^e^ Approximate integer-hour cutoffs corresponding to the tertiles were used to define the categories presented in the table. The exact tertile cutoffs were 6:30 and 7:00 for breakfast timing, 11:30 and 11:50 for lunch timing, and 17:00 and 18:00 for dinner timing.

**Table 3:** Associations of temporal eating patterns with prevalent CVD among older adults.

Temporal Eating Patterns	No.	CVD, *n* (%)	OR (95% CI)			
			Model I ^a^	Model II ^b^	Model III ^c^	Model IV ^d^
CVD cases/total N			196/1305	193/1272	192/1244	192/1243
Typical eating	758	105 (13.9)	Ref.	Ref.	Ref.	Ref.
Early-phase eating	142	12 (8.5)	0.57 (0.31, 1.07)	0.55 (0.29, 1.06)	0.55 (0.28, 1.07)	0.55 (0.28, 1.08)
Late-phase eating	405	79 (19.5)	1.51 (1.09, 2.08)	1.50 (1.06, 2.12)	1.56 (1.08, 2.26)	1.54 (1.06, 2.25)

^a^ Model I was an unadjusted model; ^b^ Model II adjusted for age, sex, education level, community type, annual household income, marital status, family history of CVD, and survey period; ^c^ Model III was the primary model and additionally adjusted for smoking status, drinking status, physical activity, diet quality, meal frequency, satiety, sleep duration, sleep quality, and shift work based on Model II. Because wake-up time and bedtime were used to derive the indicators for constructing the temporal eating patterns, chronotype was not adjusted for in Model III to avoid potential over-adjustment; ^d^ Model IV was an additional adjustment model and was further adjusted for metabolic factors, including BMI, diabetes, and hypertension based on Model III.

## Data Availability

The data presented in this study are available on request from the corresponding author due to privacy.

## Supplementary Materials

The following supporting information can be downloaded at: https://www.mdpi.com/article/10.3390/nu18172778/s1, Table S1: Model fit indices for latent profile analysis; Table S2: Meal timing variables across three temporal eating patterns; Table S3: Prevalence of prevalent CVD according to basic characteristics of older adults; Table S4: Characteristics of older adults by meal timing; Table S5: Associations of breakfast timing with history of coronary heart disease and stroke among older adults; Table S6: Associations of lunch timing with history of coronary heart disease and stroke among older adults; Table S7: Associations of dinner timing with history of coronary heart disease and stroke among older adults; Table S8: Associations of temporal eating patterns with history of coronary heart disease and stroke among older adults; Table S9: Subgroup analysis for breakfast time and prevalent CVD; Table S10: Subgroup analysis for lunch time and prevalent CVD; Table S11: Subgroup analysis for dinner time and prevalent CVD; Table S12: Subgroup analysis for temporal timing patterns and prevalent CVD; Table S13: Sensitivity analysis of the association between breakfast timing and prevalent CVD; Table S14: Sensitivity analysis of the association between lunch timing and prevalent CVD; Table S15: Sensitivity analysis of the association between dinner timing and prevalent CVD; Table S16: Sensitivity analysis of the association between temporal timing patterns and prevalent CVD; Table S17: Sensitivity analysis of the association between temporal eating patterns and prevalent CVD after excluding participants who reported late-night snacking; Figure S1: RCS for the associations of breakfast, lunch, and dinner timing with prevalent CVD; Figure S2: Curves of the sensitivity analysis for unobserved confounders with E-value highlighted.

## Abbreviations

The following abbreviations are used in this manuscript:

CVD	Cardiovascular disease
GBD	Global Burden of Disease Study
CHD	Coronary heart disease
BMI	Body mass index
IPAQ-SF	International Physical Activity Questionnaire-Short Form
MET	Metabolic equivalent of task
PSQI	Pittsburgh Sleep Quality Index
rMEQ-5	Reduced Morningness-Eveningness Questionnaire-5
VIF	Variance Inflation Factor
SD	Standard deviation
IQR	Interquartile Range
OR	Odds ratio
CI	Confidence intervals
RCS	Restricted cubic splines
LPA	Latent Profile Analysis
AIC	Akaike information criterion
BIC	Bayesian information criterion
aBIC	sample-size adjusted Bayesian information criterion
VLMR-LRT	Vuong–Lo–Mendell–Rubin likelihood ratio test
BLRT	Bootstrap likelihood ratio test
DIT	Diet-induced thermogenesis
