# Supplementary material for: Associations of Meal Timing and Temporal Eating Patterns with Cardiovascular Disease Among Chinese Older Adults
Source: Nutrients. 2026 Aug 25;18(17):2778. doi: 10.3390/nu18172778 (PMC13567632; doi:10.3390/nu18172778)
Supplement: Supplementary file 1 [file nutrients-18-02778-s001.zip › nutrients-4487928-supplementary.pdf]

Supplementary material

Associations of meal timing and temporal eating patterns with cardiovascular disease among Chinese older adults

Table S1. Model fit indices for latent profile analysis

Table S2. Meal timing variables across three temporal eating patterns

Table S3. Prevalence of CVD according to basic characteristics of older adults

Table S4. Characteristics of older adults by meal timing

Table S5. Associations of breakfast timing with history of coronary heart disease and stroke among older adults

Table S6. Associations of lunch timing with history of coronary heart disease and stroke among older adults

Table S7. Associations of dinner timing with history of coronary heart disease and stroke among older adults

Table S8. Associations of temporal eating patterns with history of coronary heart disease and stroke among older adults

Table S9. Subgroup analysis for breakfast time and prevalent CVD

Table S10. Subgroup analysis for lunch time and prevalent CVD

Table S11. Subgroup analysis for dinner time and prevalent CVD

Table S12. Subgroup analysis for temporal timing patterns and prevalent CVD

Table S13. Sensitivity analysis of the association between breakfast timing and prevalent CVD

Table S14. Sensitivity analysis of the association between lunch timing and prevalent CVD

Table S15. Sensitivity analysis of the association between dinner timing and prevalent CVD

Table S16. Sensitivity analysis of the association between temporal timing patterns and prevalent CVD

Table S17. Sensitivity analysis of the association between temporal eating patterns and prevalent CVD after excluding participants who reported late-night snacking

Fig S1 RCS for the associations of breakfast, lunch, and dinner timing with prevalent CVD

Fig S2 Curves of the sensitivity analysis for unobserved confounders with E-value highlighted

Table S1. Model fit indices for latent profile analysis

No.of classes	AIC	BIC	aBIC	Entrony	VLMR - LRT	BLRT	Sample Proportion (%) per class
2	13953.130	14020.392	13979.097	0.732	<0.001	<0.001	56.9/43.1
3	13424.382	13517.514	13460.336	0.834	<0.001	<0.001	58.1/10.9/31.0
4	12977.634	13096.635	13023.575	0.862	0.030	<0.001	29.0/55.8/11.2/4.1
5	12623.594	12768.465	12679.523	0.860	<0.001	<0.001	4.0/33.2/19.2/39.2/4.4
6	12335.317	12506.058	12401.232	0.856	0.6724	<0.001	18.3/29.3/12.8/4.7/3.6

Table S2. Meal timing variables across three temporal eating patterns

	temporal eating patterns [1]		
Characteristic	typical eating	early-phase eating	late-phase eating
Time from wake up to breakfast, h	1.0 (0.7)	0.8 (0.6)	1.4 (1.0)
Time from dinner to sleep, h	3.7 (0.7)	5.7 (0.8)	1.8 (0.8)
Eating Window, h	10.7 (1.0)	10.1 (0.8)	11.3 (1.3)
Time from eating midpoint to awake midpoint, h	1.3 (0.4)	2.4 (0.4)	0.2 (0.5)

[1] Data are presented as mean (SD).

Tabel S3. Prevalence of CVD according to basic characteristics of older adults

Characteristics	CVD		*P* value
	No (*n* = 1,109)	Yes (*n* = 196)	
Age, years			**<0.001**
<65	322 (29.0)	31 (15.8)	
65-74	560 (50.5)	88 (44.9)	
≥75	227 (20.5)	77 (39.3)	
Gender			0.343
Female, *n* (%)	612 (85.8)	101 (14.2)	
Male, *n* (%)	497 (84.0)	95 (16.0)	
Residence, *n* (%)			0.209
Urban	230 (87.5)	33 (12.6)	
Rural	879 (84.4)	163 (15.6)	
Education level, *n* (%)			0.927
Illiterate	290 (84.8)	52 (15.2)	
Primary school	480 (85.4)	82 (14.6)	
Junior high school or above	339 (84.5)	62 (15.5)	
Marital status, *n* (%)			0.099
Married	925 (82.7)	154 (14.3)	
Divorced/widowed/ never married	184 (81.4)	42 (18.6)	
Annual household income, *n* (%) [1]			**0.003**
<20,000 RMB	236 (78.7)	64 (21.3)	
20,000 - 49,999 RMB	466 (87.1)	69 (12.9)	
≥50,000 RMB	385 (86.3)	61 (13.7)	
Diabetes, *n* (%)			**<0.001**
No	956 (91.1)	148 (8.9)	
Yes	153 (78.6)	48 (21.4)	
Hypertension, *n* (%)			**<0.001**
No	606 (54.64)	59 (30.10)	
Yes	503 (45.36)	137 (69.90)	
Family history of CVD, *n* (%) [1]			**<0.001**
No	1015 (86.7)	156 (13.3)	
Yes	78 (67.2)	38 (32.8)	
Smoking status, *n* (%) [1]			0.070
Never smoker	791 (85.8)	131 (14.2)	
Former smoker	146 (79.4)	38 (20.7)	
Current smoker	170 (86.3)	27 (13.7)	
Drinking status, *n* (%) [1]			**<0.001**
Never drinker	736 (85.8)	122 (14.2)	
Former drinker	112 (71.8)	44 (28.2)	
Current drinker	259 (89.6)	30 (10.4)	
Physical activity level, *n* (%) [1]			**<0.001**
Low	167 (76.3)	52 (23.7)	

Moderate	449 (81.5)	102 (18.5)	
High	489 (92.1)	42 (7.9)	
Body mass index, *n* (%) [1]			**<0.001**
<24.00 kg/m^2	661 (87.8)	92 (12.2)	
≥24.00 kg/m^2	446 (81.1)	104 (18.9)	
Diet quality, *n* (%) [1]			0.553
Low (<4 points)	937 (84.6)	170 (15.4)	
High (≥4 points)	158 (86.3)	25 (13.7)	
Satiety [1]	7.85 ± 1.00	7.73 ± 1.06	0.139
Meal frequency [1]	3.11 ± 0.36	3.08 ± 0.35	0.228
Sleep duration, *n* (%)			**0.014**
<6 h	191 (78.9)	51 (21.1)	
6-8 h	709 (86.3)	113 (13.8)	
>8 h	209 (86.7)	32 (13.3)	
Sleep quality, *n* (%) [1]			**<0.001**
Good	824 (88.8)	104 (11.2)	
Poor	274 (75.1)	91 (24.9)	
Chronotype, *n* (%) [1]			0.665
Intermediate type	112 (84.9)	20 (15.2)	
Moderately morning type	472 (86.1)	76 (13.9)	
Definite morning type	519 (84.3)	97 (15.8)	
Shift work, *n* (%) [1]			0.131
No	914 (83.2)	172 (15.8)	
Yes	181 (88.3)	24 (11.7)	
Breakfast time [2]	6.9 ± 0.9	7.0 ± 0.8	**0.032**
Lunch time [2]	11.6 ± 0.5	11.6 ± 0.6	0.308
Dinner time [2]	17.7 ± 0.8	17.7 ± 0.8	0.345
Wake time [2]	5.8 ± 1.0	5.9 ± 1.0	0.247
Bed time [2]	21.1 ± 1.3	20.7 ± 1.4	**<0.001**

[1]Missing data: a*nn*ual household income (*n*=24, 1.84%), family history of CVD (*n*=18, 1.38%), smoking status (*n*=2, 0.15%), drinking status (*n*=2, 0.15%), physical activity level (*n*=4, 0.31%), BMI(*n*=2, 0.15%), diet quality (*n*=15, 1.15%), satiety (*n*=17, 1.3%), meal frequency (*n*=14, 1.07%), sleep quality (*n*=12, 0.92%), chronotype (*n*=9, 0.69%), shift work (*n*=14,1.07%).

[2] Time information was expressed as decimal, for example:5.50 means 5:30, 17.50 means 17:30.

Table S4. Characteristics of older adults by meal timing

Characteristics	Strata：breakfast time				Strata：lunch time				Strata：dinner time			
	≤6:00 (*n* = 283)	6:00 - 7:00 (*n* = 589)	>7:00 (*n* = 433)	*P*	≤11:00 (*n* = 366)	11:00 - 12:00 (*n* = 829)	>12:00 (*n* = 110)	*P*	≤17:00 (*n* = 461)	17:00 - 18:00 (*n* = 604)	>18:00 (*n* = 240)	*P*
Age, years	70.0 ± 6.9	69.7 ± 6.7	69.1 ± 7.1	0.082	70.6 ± 7.4	69.3 ± 6.6	68.5 ± 6.8	**0.008**	71.2 ± 7.4	69.2 ± 6.5	67.7 ± 6.1	**<0.001**
Female, *n* (%)	152 (53.7)	316 (53.7)	245 (56.6)	0.609	205 (56.0)	451 (54.4)	57 (51.8)	0.723	262 (56.8)	329 (54.5)	122 (50.8)	0.316
Residence, *n* (%)				0.225				**0.005**				**<0.001**
Urban	47 (16.6)	127 (21.6)	89 (20.6)		53 (14.5)	188 (22.7)	22 (20.0)		83 (18.0)	152 (25.2)	28 (11.7)	
Rural	236 (83.4)	462 (78.4)	344 (79.4)		313 (85.5)	641 (77.3)	88 (80.0)		378 (82.0)	452 (74.8)	212 (88.3)	
Education level, *n* (%)				0.266				0.346				**0.002**
Illiterate	81 (28.6)	151 (25.6)	110 (25.4)		106 (29.0)	204 (24.6)	32 (29.1)		148 (32.1)	138 (22.8)	56 (23.3)	
Primary school	130 (45.9)	253 (43.0)	179 (41.3)		160 (43.7)	358 (43.2)	44 (40.0)		196 (42.5)	259 (42.9)	107 (44.6)	
Junior high school or above	72 (25.4)	185 (31.4)	144 (33.3)		100 (27.3)	267 (32.2)	34 (30.9)		117 (25.4)	207 (34.3)	77 (32.1)	
Marital status, *n* (%)				0.399				0.549				**0.010**
Married	229 (80.9)	496 (84.2)	354 (81.8)		296 (80.9)	692 (83.5)	91 (82.7)		362 (78.5)	517 (85.6)	200 (83.3)	
Divorced/widowed/ never married	54 (19.1)	93 (15.8)	79 (18.2)		70 (19.1)	137 (16.5)	19 (17.3)		99 (21.5)	87 (14.4)	40 (16.7)	
Annual household income, *n* (%) [1]				**0.002**				**0.002**				**<0.001**
<20,000 RMB	66 (23.8)	125 (21.6)	109 (25.6)		74 (20.6)	184 (22.6)	42 (38.5)		85 (18.8)	136 (22.9)	79 (33.5)	
20,000 - 49,999 RMB	97 (35.0)	241 (41.6)	197 (46.4)		155 (43.2)	339 (41.7)	41 (37.6)		235 (52.0)	217 (36.6)	83 (35.2)	
≥50,000 RMB	114 (41.2)	213 (36.8)	119 (28.0)		130 (36.2)	290 (35.7)	26 (23.9)		132 (29.2)	240 (40.5)	74 (31.4)	

Diabetes, *n* (%)				0.285				0.552				**0.024**
No	247 (87.3)	498 (84.6)	359 (82.9)		308 (84.2)	699 (84.3)	97 (88.2)		373 (80.9)	522 (86.4)	209 (87.1)	
Yes	36 (12.7)	91 (15.4)	74 (17.1)		58 (15.8)	130 (15.7)	13 (11.8)		88 (19.1)	82 (13.6)	31 (12.9)	
Hypertension, *n* (%)				0.525				0.245				0.465
No	149 (52.7)	290 (49.2)	226 (52.2)		175 (47.8)	437 (52.7)	53 (48.2)		227 (49.2)	308 (51.0)	130 (54.2)	
Yes	134 (47.3)	299 (50.8)	207 (47.8)		191 (52.2)	392 (47.3)	57 (51.8)		234 (50.8)	296 (49.0)	110 (45.8)	
Family history of CVD, *n* (%) [1]				0.163				0.505				0.578
No	261 (93.9)	526 (90.2)	384 (90.1)		336 (92.3)	737 (90.7)	98 (89.1)		409 (89.9)	544 (91.4)	218 (92.0)	
Yes	17 (6.1)	57 (9.8)	42 (9.9)		28 (7.7)	76 (9.3)	12 (10.9)		46 (10.1)	51 (8.6)	19 (8.0)	
Smoking status, *n* (%) [1]				0.836				0.219				**0.009**
Never smoker	195 (69.1)	417 (70.9)	310 (71.6)		255 (69.9)	599 (72.3)	68 (61.8)		338 (73.5)	431 (71.5)	153 (63.7)	
Former smoker	44 (15.6)	85 (14.5)	55 (12.7)		52 (14.2)	113 (13.6)	19 (17.3)		55 (12.0)	94 (15.6)	35 (14.6)	
Current smoker	43 (15.2)	86 (14.6)	68 (15.7)		58 (15.9)	116 (14.0)	23 (20.9)		67 (14.6)	78 (12.9)	52 (21.7)	
Drinking status, *n* (%) [1]				**0.048**				**0.001**				0.157
Never drinker	166 (58.7)	394 (67.0)	298 (69.0)		209 (57.1)	577 (69.8)	72 (65.5)		303 (65.9)	400 (66.3)	155 (64.6)	
Former drinker	41 (14.5)	72 (12.2)	43 (10.0)		57 (15.6)	86 (10.4)	13 (11.8)		64 (13.9)	71 (11.8)	21 (8.8)	
Current drinker	76 (26.9)	122 (20.7)	91 (21.1)		100 (27.3)	164 (19.8)	25 (22.7)		93 (20.2)	132 (21.9)	64 (26.7)	
Physical activity level, *n* (%) [1]				0.364				0.178				0.079
Low	46 (16.3)	104 (17.7)	69 (16.0)		74 (20.2)	124 (15.0)	21 (19.4)		83 (18.0)	95 (15.8)	41 (17.2)	
Moderate	113 (39.9)	261 (44.5)	177 (41.0)		154 (42.1)	351 (42.4)	46 (42.6)		179 (38.8)	280 (46.6)	92 (38.5)	
High	124 (43.8)	221 (37.7)	186 (43.1)		138 (37.7)	352 (42.6)	41 (38.0)		199 (43.2)	226 (37.6)	106 (44.4)	
Body mass index, *n* (%) [1]				0.664				0.627				0.315

<24.00 kg/m²	164 (58.0)	346 (58.9)	243 (56.1)		208 (57.0)	486 (58.6)	59 (54.1)		274 (59.6)	335 (55.6)	144 (60.0)	
≥24.00 kg/m²	119 (42.0)	241 (41.1)	190 (43.9)		157 (43.0)	343 (41.4)	50 (45.9)		186 (40.4)	268 (44.4)	96 (40.0)	
Diet quality, *n* (%) [1]				0.149				0.07				**0.049**
Low (<4 points)	246 (89.5)	496 (84.8)	365 (84.9)		304 (84.0)	701 (85.7)	102 (92.7)		383 (84.4)	507 (84.9)	217 (90.8)	
High (≥4 points)	29 (10.5)	89 (15.2)	65 (15.1)		58 (16.0)	117 (14.3)	8 (7.3)		71 (15.6)	90 (15.1)	22 (9.2)	
Satiety [1]	7.9 ± 1.1	7.8 ± 1.0	7.8 ± 1.0	0.374	7.8 ± 1.0	7.8 ± 1.0	7.8 ± 0.9	0.887	7.7 ± 1.1	7.9 ± 1.0	8.0 ± 1.0	**0.005**
Meal frequency [1]	3.1 ± 0.3	3.1 ± 0.3	3.1 ± 0.4	0.504	3.1 ± 0.4	3.1 ± 0.3	3.1 ± 0.3	0.088	3.1 ± 0.4	3.1 ± 0.3	3.1 ± 0.3	**0.031**
Sleep duration, *n* (%)				**<0.001**				0.058				**<0.001**
<6 h	61 (21.6)	92 (15.6)	89 (20.6)		71 (19.4)	144 (17.4)	27 (24.5)		83 (18.0)	94 (15.6)	65 (27.1)	
6-8 h	189 (66.8)	388 (65.9)	245 (56.6)		223 (60.9)	526 (63.4)	73 (66.4)		257 (55.7)	417 (69.0)	148 (61.7)	
>8 h	33 (11.7)	109 (18.5)	99 (22.9)		72 (19.7)	159 (19.2)	10 (9.1)		121 (26.2)	93 (15.4)	27 (11.2)	
Sleep quality, *n* (%) [1]				**0.017**				**0.009**				**0.020**
Good	210 (75.0)	431 (73.9)	287 (66.7)		255 (70.6)	607 (73.8)	66 (60.0)		335 (73.8)	439 (73.2)	154 (64.4)	
Poor	70 (25.0)	152 (26.1)	143 (33.3)		106 (29.4)	215 (26.2)	44 (40.0)		119 (26.2)	161 (26.8)	85 (35.6)	
Chronotype, *n* (%) [1]				**<0.001**				**<0.001**				**<0.001**
Intermediate type	9 (3.2)	53 (9.1)	70 (16.3)		21 (5.8)	88 (10.7)	23 (20.9)		32 (7.0)	62 (10.3)	38 (15.9)	
Moderately morning type	110 (39.0)	247 (42.2)	191 (44.5)		126 (34.9)	374 (45.3)	48 (43.6)		181 (39.8)	257 (42.7)	110 (46.0)	
Definite morning type	163 (57.8)	285 (48.7)	168 (39.2)		214 (59.3)	363 (44.0)	39 (35.5)		242 (53.2)	283 (47.0)	91 (38.1)	
Shift work, *n* (%) [1]				0.711				**0.048**				0.260
No	237 (84.9)	494 (84.6)	355 (82.9)		295 (81.3)	691 (84.5)	100 (90.9)		384 (84.0)	493 (82.9)	209 (87.4)	
Yes	42 (15.1)	90 (15.4)	73 (17.1)		68 (18.7)	127 (15.5)	10 (9.1)		73 (16.0)	102 (17.1)	30 (12.6)	

[1] Missing data：a*nn*ual household income (*n*=24, 1.84%), family history of CVD (*n*=18, 1.38%), smoking status (*n*=2, 0.15%), drinking status (*n*=2, 0.15%), physical activity level (*n*=4, 0.31%), BMI(*n*=2, 0.15%), diet quality (*n*=15, 1.15%), satiety (*n*=17, 1.3%), meal frequency (*n*=14, 1.07%), sleep quality (*n*=12, 0.92%), chronotype (*n*=9, 0.69%), shift work (*n*=14,1.07%).

Table S5. Associations of breakfast timing with history of coronary heart disease and stroke among older adults

Outcome	No.	*n* (%)	OR (95%CI)			
			Model I [a]	Model II [b]	Model III [c]	Model IV [d]
Coronary heart disease [e]						
Continuous	1305	121 (9.3)	**1.25 (1.00, 1.55)**	**1.30 (1.03, 1.65)**	1.20 (0.92, 1.57)	1.18 (0.90, 1.54)
Categorical [f]						
≤6:00	283	20 (7.1)	Ref.	Ref.	Ref.	Ref.
6:00 - 7:00	589	52 (8.8)	1.27 (0.74, 2.18)	1.31 (0.75, 2.31)	1.16 (0.64, 2.11)	1.13 (0.62, 2.06)
>7:00	433	49 (11.3)	1.68 (0.97, 2.89)	**1.87 (1.04, 3.37)**	1.60 (0.84, 3.05)	1.57 (0.82, 3.00)
P for trend			**0.050**	**0.029**	0.120	0.136
Stroke [e]						
Continuous	1305	96 (7.4)	1.22 (0.96, 1.56)	1.28 (0.99, 1.66)	**1.43 (1.06, 1.93)**	**1.38 (1.01, 1.88)**
Categorical [f]						
≤6:00	283	16 (5.7)	Ref.	Ref.	Ref.	Ref.
6:00 - 7:00	589	44 (7.5)	1.35 (0.75, 2.43)	1.55 (0.83, 2.90)	1.65 (0.83, 3.28)	1.49 (0.74, 2.98)
>7:00	433	36 (8.3)	1.51 (0.82, 2.78)	1.78 (0.92, 3.42)	2.07 (0.99, 4.34)	1.91 (0.90, 4.06)
P for trend			0.195	0.100	0.060	0.093

[a] Model I was an unadjusted model.

[b] Model II adjusted for age, sex, education level, community type, annual household income, marital status, family history of CVD, and survey period.

[c] Model III was the primary model and additionally adjusted for smoking status, drinking status, IPAQ level, dietary quality, meal frequency, satiety, sleep duration, sleep quality, chronotype, and shift work, based on Model II.

[d] Model IV was an additional adjustment model and was further adjusted for metabolic factors, including BMI, diabetes, and hypertension, based on Model III.

[e] Twenty-one participants had both CHD and stroke, and 2 participants had both CHD and heart failure.

[f] Approximate integer-hour cutoffs corresponding to the tertiles were used to define the categories presented in the table. The exact tertile cutoffs were 6:30 and 7:00 for breakfast timing.

Table S6. Associations of lunch timing with history of coronary heart disease and stroke among older adults

Outcome	No.	*n* (%)	OR (95%CI)			
			Model I [a]	Model II [b]	Model III [c]	Model IV [d]
Coronary heart disease [e]						
Continuous	1305	121 (9.3)	1.34 (0.97, 1.86)	**1.55 (1.09, 2.22)**	**1.47 (1.00, 2.15)**	**1.51 (1.02, 2.23)**
Categorical [f]						
≤11:00	366	29 (7.9)	Ref.	Ref.	Ref.	Ref.
11:00 - 12:00	829	78 (9.4)	1.21 (0.77, 1.88)	1.40 (0.87, 2.25)	1.46 (0.88, 2.42)	1.47 (0.88, 2.45)
>12:00	110	14 (12.7)	1.69 (0.86, 3.33)	**2.17 (1.03, 4.58)**	1.82 (0.81, 4.06)	1.95 (0.86, 4.41)
P for trend			0.145	**0.039**	0.094	0.074
Stroke [e]						
Continuous	1305	96 (7.4)	1.04 (0.72, 1.52)	1.11 (0.74, 1.65)	1.17 (0.76, 1.80)	1.24 (0.80, 1.93)
Categorical [f]						
≤11:00	366	26 (7.1)	Ref.	Ref.	Ref.	Ref.
11:00 - 12:00	829	59 (7.1)	1.00 (0.62, 1.62)	1.09 (0.66, 1.79)	1.34 (0.77, 2.32)	1.36 (0.78, 2.39)
>12:00	110	11 (10.0)	1.45 (0.69, 3.04)	1.58 (0.71, 3.49)	1.62 (0.68, 3.88)	1.93 (0.79, 4.73)
P for trend			0.477	0.346	0.223	0.134

[a] Model I was an unadjusted model.

[b] Model II adjusted for age, sex, education level, community type, annual household income, marital status, family history of CVD, and survey period.

[c] Model III was the primary model and additionally adjusted for smoking status, drinking status, IPAQ level, dietary quality, meal frequency, satiety, sleep duration, sleep quality, chronotype, and shift work, based on Model II.

[d] Model IV was an additional adjustment model and was further adjusted for metabolic factors, including BMI, diabetes, and hypertension, based on Model III.

[e] Twenty-one participants had both CHD and stroke, and 2 participants had both CHD and heart failure.

[f] Approximate integer-hour cutoffs corresponding to the tertiles were used to define the categories presented in the table. The exact tertile cutoffs were 11:30 and 11:50 for lunch timing.

Table S7. Associations of dinner timing with history of coronary heart disease and stroke among older adults

Outcome	No.	*n* (%)	OR (95%CI)			
			Model I [a]	Model II [b]	Model III [c]	Model IV [d]
Coronary heart disease [e]						
Continuous	1305	121 (9.3)	1.09 (0.87, 1.36)	**1.41 (1.09, 1.84)**	**1.54 (1.15, 2.06)**	**1.58 (1.18, 2.13)**
Categorical [f]						
≤17:00	461	35 (7.6)	Ref.	Ref.	Ref.	Ref.
17:00-18:00	604	60 (9.9)	1.34 (0.87, 2.08)	**1.65 (1.02, 2.66)**	1.57 (0.95, 2.60)	1.63 (0.98, 2.71)
>18:00	240	26 (10.8)	1.48 (0.87, 2.52)	**2.43 (1.32, 4.45)**	**2.55 (1.33, 4.91)**	**2.87 (1.47, 5.58)**
P for trend			0.125	**0.003**	**0.005**	**0.002**
Stroke [e]						
Continuous	1305	96 (7.4)	1.09 (0.85, 1.40)	1.21 (0.91, 1.60)	1.32 (0.96, 1.81)	**1.41 (1.02, 1.96)**
Categorical [f]						
≤17:00	461	30 (6.5)	Ref.	Ref.	Ref.	Ref.
17:00-18:00	604	40 (6.6)	1.02 (0.62, 1.66)	1.09 (0.65, 1.84)	1.15 (0.65, 2.03)	1.30 (0.73, 2.32)
>18:00	240	26 (10.8)	**1.75 (1.01, 3.03)**	**2.30 (1.24, 4.27)**	**2.90 (1.44, 5.83)**	**3.84 (1.85, 7.97)**
P for trend			0.069	**0.015**	**0.007**	**<0.001**

[a] Model I was an unadjusted model.

[b] Model II adjusted for age, sex, education level, community type, annual household income, marital status, family history of CVD, and survey period.

[c] Model III was the primary model and additionally adjusted for smoking status, drinking status, IPAQ level, dietary quality, meal frequency, satiety, sleep duration, sleep quality, chronotype, and shift work, based on Model II.

[d] Model IV was an additional adjustment model and was further adjusted for metabolic factors, including BMI, diabetes, and hypertension, based on Model III.

[e] Twenty-one participants had both CHD and stroke, and 2 participants had both CHD and heart failure.

[f] Approximate integer-hour cutoffs corresponding to the tertiles were used to define the categories presented in the table. The exact tertile cutoffs were 17:00 and 18:00 for dinner timing.

Table S8. Associations of temporal eating patterns with history of coronary heart disease and stroke among older adults

Outcome	No.	*n* (%)	OR (95%CI)			
			Model I [a]	Model II [b]	Model III [c]	Model IV [d]
Coronary heart disease [e]						
Typical eating	758	71 (9.4)	Ref.	Ref.	Ref.	Ref.
Early-phase eating	142	6 (4.3)	**0.43 (0.18, 1.00)**	**0.40 (0.17, 0.96)**	**0.37 (0.15, 0.91)**	**0.37 (0.15, 0.90)**
Late-phase eating	405	44 (10.9)	1.18 (0.79, 1.75)	1.21 (0.79, 1.86)	1.22 (0.78, 1.91)	1.20 (0.76, 1.89)
Stroke [e]						
Typical eating	758	43 (5.7)	Ref.	Ref.	Ref.	Ref.
Early-phase eating	142	6 (4.2)	0.73 (0.31, 1.76)	0.72 (0.30, 1.75)	0.75 (0.30, 1.88)	0.76 (0.30, 1.93)
Late-phase eating	405	47 (11.6)	**2.18 (1.42, 3.36)**	**1.98 (1.25, 3.13)**	**2.08 (1.28, 3.38)**	**2.04 (1.25, 3.34)**

[a] Model I was an unadjusted model.

[b] Model II adjusted for age, sex, education level, community type, annual household income, marital status, family history of CVD, and survey period.

[c] Model III was the primary model and additionally adjusted for smoking status, drinking status, IPAQ level, dietary quality, meal frequency, satiety, sleep duration, sleep quality, and shift work, based on Model II. Because wake-up time and bedtime were used to derive the indicators for constructing the temporal eating patterns, chronotype was not adjusted for in Model III to avoid potential over-adjustment.

[d] Model IV was an additional adjustment model and was further adjusted for metabolic factors, including BMI, diabetes, and hypertension, based on Model III.

[e] Twenty-one participants had both CHD and stroke, and 2 participants had both CHD and heart failure.

Table S9. Subgroup analysis for breakfast time and prevalent CVD

**Subgroup**	***n***	OR (95%CI)			***P*** ***interaction***
		≤6:00 (*n* = 283)	6:00 - 7:00 (*n* = 589)	>7:00 (*n* = 433)	
**Age**					0.738
≤70 years	752	Ref	1.67 (0.73, 3.80)	1.88 (0.80, 4.44)	
>70 years	553	Ref	1.29 (0.67, 2.47)	1.71 (0.81, 3.58)	
**Gender**					0.520
Male	592	Ref	1.65 (0.77, 3.50)	2.27 (0.99, 5.21)	
Female	713	Ref	1.20 (0.62, 2.35)	1.45 (0.70, 3.00)	
**Smoking status**					0.671
Never	922	Ref	1.48 (0.81, 2.70)	1.67 (0.87, 3.22)	
Past and current	381	Ref	1.28 (0.51, 3.18)	2.17 (0.79, 5.96)	
**Drinking status**					0.947
Never	858	Ref	1.37 (0.70, 2.67)	1.64 (0.81, 3.33)	
Past and current	445	Ref	1.44 (0.68, 3.07)	1.80 (0.77, 4.22)	
**Physical activity**					0.760
Low/Moderate	770	Ref	1.60 (0.90, 2.82)	**2.03 (1.08, 3.82)**	
High	531	Ref	1.24 (0.46, 3.37)	1.11 (0.39, 3.16)	
**Body Mass Index**					0.594
<24.00 kg/m^2	753	Ref	2.02 (0.91, 4.51)	**2.76 (1.13, 6.73)**	
≥24.00 kg/m^2	550	Ref	1.09 (0.55, 2.16)	1.18 (0.56, 2.45)	
**Sleep duration**					0.905
6-8h	822	Ref	1.52 (0.80, 2.87)	1.78 (0.87, 3.66)	
<6h/>8h	483	Ref	1.27 (0.56, 2.88)	1.77 (0.75, 4.15)	
**Sleep quality**					0.768
Good	928	Ref	1.70 (0.88, 3.26)	**2.12 (1.04, 4.34)**	
bad	365	Ref	1.22 (0.55, 2.69)	1.53 (0.64, 3.65)	

The model was adjusted for the same covariates as Model III, except that the corresponding stratification variable was not included in each subgroup analysis.

Table S10. Subgroup analysis for lunch time and prevalent CVD

**Subgroup**	***n***	OR (95%CI)			***P*** ***interaction***
		≤11:00 (*n* = 366)	11:00 - 12:00 (*n* = 829)	>12:00 (*n* = 110)	
**Age**					0.719
≤70 years	752	Ref	1.59 (0.84, 3.01)	1.62 (0.56, 4.65)	
>70 years	553	Ref	1.07 (0.61, 1.85)	1.16 (0.45, 2.97)	
**Gender**					0.403
Male	592	Ref	**2.16 (1.11, 4.19)**	1.96 (0.68, 5.66)	
Female	713	Ref	1.09 (0.63, 1.90)	1.55 (0.61, 3.92)	
**Smoking status**					0.523
Never	922	Ref	1.21 (0.74, 1.97)	1.34 (0.57, 3.12)	
Past and current	381	Ref	**2.76 (1.19, 6.36)**	3.50 (0.98, 12.45)	
**Drinking status**					0.449
Never	858	Ref	1.33 (0.77, 2.29)	1.73 (0.75, 4.01)	
Past and current	445	Ref	1.69 (0.88, 3.26)	1.13 (0.33, 3.83)	
**Physical activity**					0.565
Low/Moderate	770	Ref	1.50 (0.93, 2.42)	1.82 (0.85, 3.87)	
High	531	Ref	1.08 (0.45, 2.60)	0.85 (0.15, 4.98)	
**Body Mass Index**					0.378
<24.00 kg/m^2	753	Ref	1.07 (0.57, 1.99)	1.42 (0.52, 3.83)	
≥24.00 kg/m^2	550	Ref	1.83 (0.98, 3.41)	1.85 (0.64, 5.35)	
**Sleep duration**					0.502
6-8h	822	Ref	1.29 (0.75, 2.21)	1.16 (0.46, 2.92)	
<6h/>8h	483	Ref	1.62 (0.84, 3.10)	2.27 (0.77, 6.65)	
**Sleep quality**					0.311
Good	928	Ref	**1.91 (1.08, 3.36)**	1.33 (0.44, 4.06)	
bad	365	Ref	1.07 (0.57, 2.02)	1.39 (0.54, 3.60)	

The model was adjusted for the same covariates as Model III, except that the corresponding stratification variable was not included in each subgroup analysis.

Table S11. Subgroup analysis for dinner time and prevalent CVD

Subgroup	*n*	OR (95%CI)			*P* *interaction*
		≤17:00 (*n* = 461)	17:00 - 18:00 (*n* = 604)	>18:00 (*n* = 240)	
**Age**					0.773
≤70 years	752	Ref	0.97 (0.51, 1.86)	**2.38 (1.12, 5.08)**	
>70 years	553	Ref	1.27 (0.74, 2.18)	**2.60 (1.19, 5.69)**	
**Gender**					**0.015**
Male	592	Ref	**2.64 (1.34, 5.19)**	**3.49 (1.45, 8.42)**	
Female	713	Ref	0.70 (0.40, 1.23)	**2.25 (1.14, 4.47)**	
**Smoking status**					0.064
Never	922	Ref	0.87 (0.53, 1.42)	**2.15 (1.16, 3.99)**	
Past and current	381	Ref	**3.93 (1.60, 9.68)**	**6.63 (2.07, 21.22)**	
**Drinking status**					0.853
Never	858	Ref	1.09 (0.64, 1.84)	**2.34 (1.20, 4.56)**	
Past and current	445	Ref	1.18 (0.61, 2.28)	2.31 (0.96, 5.59)	
**Physical activity**					0.174
Low/Moderate	770	Ref	0.98 (0.61, 1.57)	2.35 (1.29, 4.28)	
High	531	Ref	**2.88 (1.12, 7.43)**	**4.86 (1.47, 16.09)**	
**Body Mass Index**					0.595
<24.00 kg/m^2	753	Ref	1.05 (0.56, 2.00)	**3.02 (1.39, 6.57)**	
≥24.00 kg/m^2	550	Ref	1.37 (0.75, 2.49)	**2.51 (1.11, 5.68)**	
**Sleep duration**					0.555
6-8h	822	Ref	1.15 (0.66, 2.00)	**2.78 (1.36, 5.69)**	
<6h/>8h	483	Ref	1.36 (0.70, 2.62)	**2.61 (1.13, 6.00)**	
**Sleep quality**					0.513
Good	928	Ref	1.54 (0.87, 2.70)	**3.08 (1.47, 6.46)**	
bad	365	Ref	0.90 (0.47, 1.71)	**2.43 (1.04, 5.70)**	

The model was adjusted for the same covariates as Model III, except that the corresponding stratification variable was not included in each subgroup analysis.

Table S12. Subgroup analysis for temporal eating patterns and prevalent CVD

**Subgroup**	***n***	OR (95%CI)			***P*** ***interaction***
		typical eating (*n* =758)	early-phase eating (*n* = 142)	late-phase eating (*n* = 405)	
**Age**					0.330
≤70 years	752	Ref	0.85 (0.35, 2.05)	**1.78 (1.02, 3.13)**	
>70 years	553	Ref	**0.28 (0.09, 0.87)**	1.41 (0.84, 2.35)	
**Gender**					0.796
Male	592	Ref	0.62 (0.23, 1.66)	1.53 (0.89, 2.63)	
Female	713	Ref	0.43 (0.17, 1.12)	1.65 (0.97, 2.80)	
**Smoking status**					0.530
Never	922	Ref	0.65 (0.30, 1.44)	**1.82 (1.15, 2.87)**	
Past and current	381	Ref	0.28 (0.07, 1.10)	1.24 (0.64, 2.40)	
**Drinking status**					0.439
Never	858	Ref	0.60 (0.26, 1.38)	**1.76 (1.09, 2.85)**	
Past and current	445	Ref	0.60 (0.19, 1.89)	1.10 (0.61, 2.00)	
**Physical activity**					0.697
Low/Moderate	770	Ref	0.57 (0.27, 1.22)	1.47 (0.95, 2.25)	
High	531	Ref	0.40 (0.08, 2.05)	**2.37 (1.10, 5.09)**	
**Body Mass Index**					0.851
<24.00 kg/m^2	753	Ref	0.42 (0.13, 1.34)	**1.79 (1.02, 3.14)**	
≥24.00 kg/m^2	550	Ref	0.71 (0.29, 1.71)	1.55 (0.90, 2.68)	
**Sleep duration**					**0.002**
6-8h	822	Ref	1.23 (0.57, 2.67)	1.41 (0.85, 2.35)	
<6h/>8h	483	Ref	**0.07 (0.01, 0.55)**	**1.83 (1.02, 3.28)**	
**Sleep quality**					0.091
Good	928	Ref	0.78 (0.35, 1.73)	1.28 (0.76, 2.15)	
bad	365	Ref	**0.31 (0.08, 1.12)**	**2.26 (1.26, 4.05)**	

The model was adjusted for the same covariates as Model III, except that the corresponding stratification variable was not included in each subgroup analysis.

Table S13. Sensitivity analysis of the association between breakfast timing and prevalent CVD

Limited population	No.	CVD, ***n*** (%)	OR (95%CI)[1]		
			≤6:00	6:00 - 7:00	>7:00
Moderately morning and Definitely morning	1,164	173 (14.86)	Ref.	1.49 (0.89, 2.48)	**1.97 (1.13, 3.46)**
No shift work history	1,086	172 (15.84)	Ref.	**1.85 (1.07, 3.21)**	**2.00 (1.09, 3.66)**
No family history of CVD	1,171	156 (13.32)	Ref.	**1.76 (1.03, 3.02)**	**2.03 (1.13, 3.65)**

[1] The model was adjusted for age (years), sex (male, female), education level (illiterate, primary school or below, junior high school or above), community type (urban, rural), annual household income (<20,000 RMB, 20,000-49,999 RMB, ≥50,000 RMB), marital status (married, unmarried), family history of CVD (yes, no), survey period (year), smoking status (never, former, current), drinking status (never, former, current), IPAQ level (low, moderate, high), dietary quality (good, poor), meal frequency (continuous), satiety (continuous), sleep duration (<6 h, 6-8 h, >8 h), sleep quality (good, poor), chronotype (definite morning type, moderate morning type, intermediate type), and shift work history (yes, no).

Table S14. Sensitivity analysis of the association between lunch timing and prevalent CVD

Limited population	No.	CVD, ***n*** (%)	OR (95%CI)[1]		
			≤11:00	11:00 - 12:00	>12:00
Moderately morning and Definitely morning	1,164	173 (14.86)	Ref.	1.53 (0.99, 2.36)	1.81 (0.86, 3.80)
No shift work history	1,086	172 (15.84)	Ref.	1.31 (0.84, 2.05)	1.50 (0.72, 3.11)
No family history of CVD	1,171	156 (13.32)	Ref.	1.41 (0.90, 2.21)	1.84 (0.87, 3.88)

[1] The model was adjusted for age (years), sex (male, female), education level (illiterate, primary school or below, junior high school or above), community type (urban, rural), annual household income (<20,000 RMB, 20,000-49,999 RMB, ≥50,000 RMB), marital status (married, unmarried), family history of CVD (yes, no), survey period (year), smoking status (never, former, current), drinking status (never, former, current), IPAQ level (low, moderate, high), dietary quality (good, poor), meal frequency (continuous), satiety (continuous), sleep duration (<6 h, 6-8 h, >8 h), sleep quality (good, poor), chronotype (definite morning type, moderate morning type, intermediate type), and shift work history (yes, no).

Table S15. Sensitivity analysis of the association between dinner timing and prevalent CVD

Limited population	No.	CVD, ***n*** (%)	OR (95%CI)[1]		
			≤17:00	17:00 - 18:00	>18:00
Moderately morning and Definitely morning	1,164	173 (14.86)	Ref.	1.49 (0.96, 2.32)	**3.11 (1.73, 5.59)**
No shift work history	1,086	172 (15.84)	Ref.	0.98 (0.62, 1.54)	**2.58 (1.45, 4.59)**
No family history of CVD	1,171	156 (13.32)	Ref.	1.30 (0.82, 2.05)	**3.04 (1.70, 5.43)**

[1] The model was adjusted for age (years), sex (male, female), education level (illiterate, primary school or below, junior high school or above), community type (urban, rural), annual household income (<20,000 RMB, 20,000-49,999 RMB, ≥50,000 RMB), marital status (married, unmarried), family history of CVD (yes, no), survey period (year), smoking status (never, former, current), drinking status (never, former, current), IPAQ level (low, moderate, high), dietary quality (good, poor), meal frequency (continuous), satiety (continuous), sleep duration (<6 h, 6-8 h, >8 h), sleep quality (good, poor), chronotype (definite morning type, moderate morning type, intermediate type), and shift work history (yes, no).

Table S16. Sensitivity analysis of the association between temporal eating patterns and prevalent CVD

Limited population	No.	CVD, ***n*** (%)	OR (95%CI)[1]		
			typical eating	early-phase eating	late-phase eating
Moderately morning and Definitely morning	1,164	173 (14.86)	Ref.	**0.40 (0.18, 0.90)**	**1.51 (1.01, 2.25)**
No shift work history	1,086	172 (15.84)	Ref.	0.56 (0.27, 1.15)	**1.74 (1.16, 2.59)**
No family history of CVD	1,171	156 (13.32)	Ref.	0.50 (0.22, 1.11)	**1.66 (1.11, 2.48)**

[1] The model was adjusted for age (years), sex (male, female), education level (illiterate, primary school or below, junior high school or above), community type (urban, rural), annual household income (<20,000 RMB, 20,000-49,999 RMB, ≥50,000 RMB), marital status (married, unmarried), family history of CVD (yes, no), survey period (year), smoking status (never, former, current), drinking status (never, former, current), IPAQ level (low, moderate, high), dietary quality (good, poor), meal frequency (continuous), satiety (continuous), sleep duration (<6 h, 6-8 h, >8 h), sleep quality (good, poor), and shift work history (yes, no).

Table S17. Sensitivity analysis for the association between temporal eating patterns and prevalent CVD after excluding participants who reported late-night snacking

Temporal eating patterns	No.	CVD, ***n*** (%)	OR (95% CI)			
			Model I [a]	Model II [b]	Model III [c]	Model IV [d]
Typical eating	751	103 (13.7)	1.00	1.00	1.00	1.00
Early-phase eating	132	11 (8.3)	0.57 (0.31,1.10)	0.55 (0.28, 1.08)	0.55 (0.28, 1.10)	0.55 (0.27, 1.10)
Late-phase eating	403	78 (19.4)	**1.51 (1.09, 2.09)**	**1.50 (1.04, 2.10)**	**1.56 (1.06, 2.23)**	**1.51 (1.04, 2.21)**

[a] Model I was an unadjusted model.

[b] Model II adjusted for age, sex, education level, community type, annual household income, marital status, family history of CVD, and survey period.

[c] Model III was the primary model and additionally adjusted for smoking status, drinking status, IPAQ level, dietary quality, meal frequency, satiety, sleep duration, sleep quality, and shift work, based on Model II. Because wake-up time and bedtime were used to derive the indicators for constructing the temporal eating patterns, chronotype was not adjusted for in Model III to avoid potential over-adjustment.

[d] Model IV was an additional adjustment model and was further adjusted for metabolic factors, including BMI, diabetes, and hypertension, based on Model III.

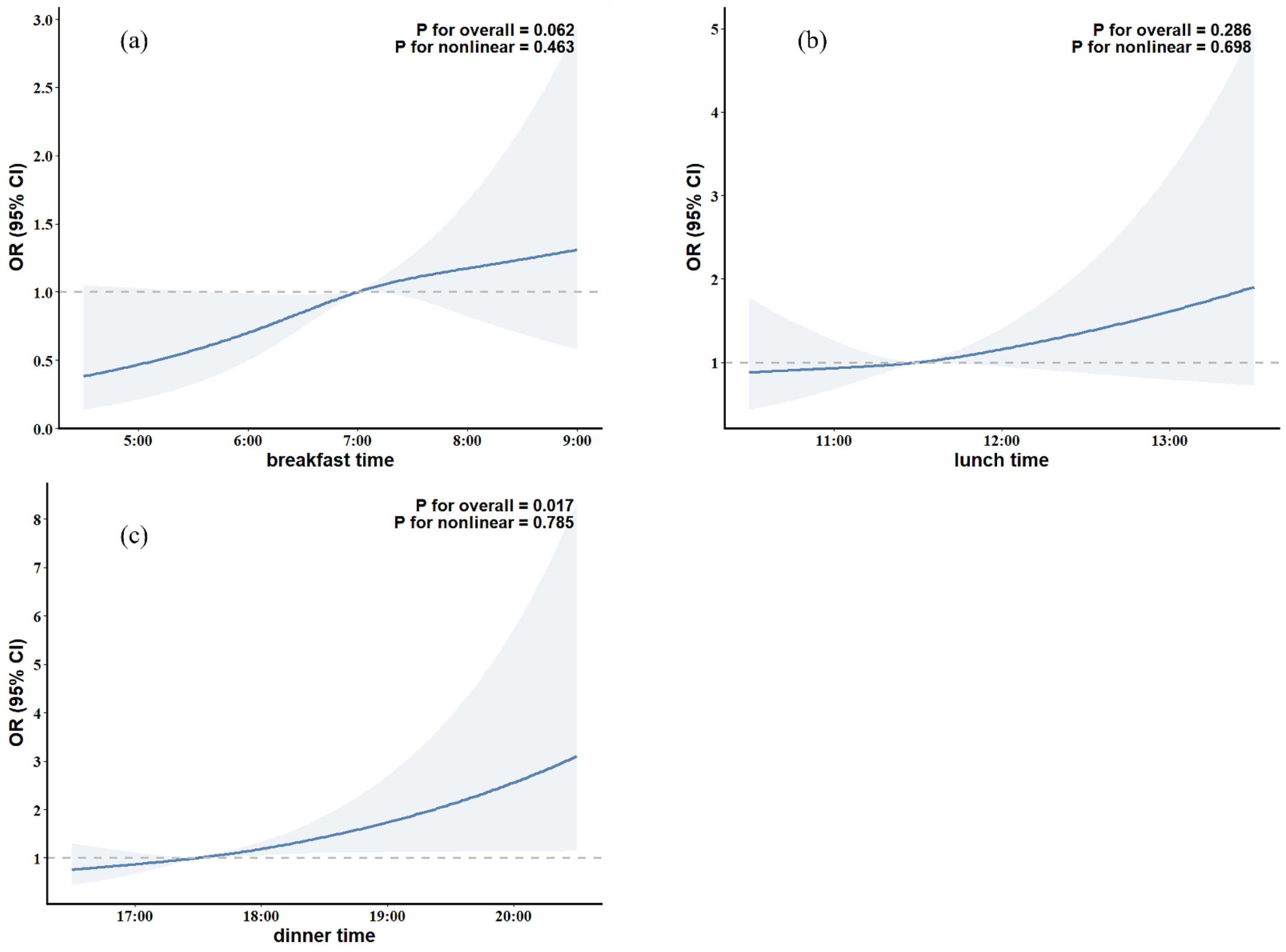

Fig S1 RCS for the associations of breakfast, lunch, and dinner Timing with prevalent CVD, all the same covariates as in Model III were adjusted.

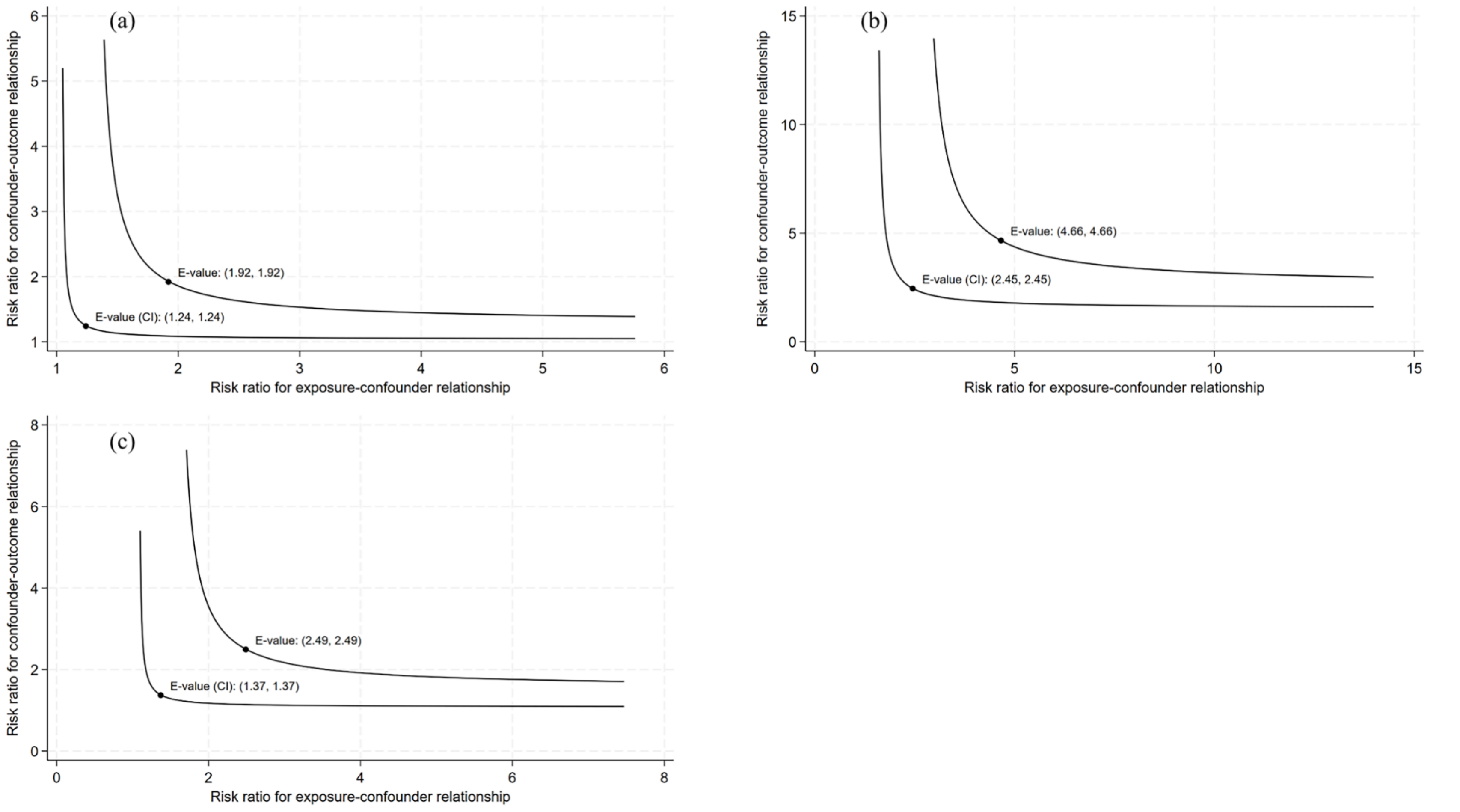

Fig S2 Curves of the sensitivity analysis for unobserved confounders with E-value highlighted (breakfast timing, dinner timing and late-phase eating)
